# Development of an Umami Taste Sensitivity Test and Its Clinical Use

**DOI:** 10.1371/journal.pone.0095177

**Published:** 2014-04-18

**Authors:** Shizuko Satoh-Kuriwada, Misako Kawai, Masahiro Iikubo, Yuki Sekine-Hayakawa, Noriaki Shoji, Hisayuki Uneyama, Takashi Sasano

**Affiliations:** 1 Division of Oral Diagnosis, Department of Oral Medicine and Surgery, Tohoku University Graduate School of Dentistry, Aoba-ku, Sendai, Japan; 2 Institute for Innovation, Ajinomoto Company Inc., Kawasaki-ku, Kawasaki, Japan; National Institute of Agronomic Research, France

## Abstract

There is a close relationship between perception of umami, which has become recognized as the fifth taste, and the human physical condition. We have developed a clinical test for umami taste sensitivity using a filter paper disc with a range of six monosodium glutamate (MSG) concentrations. We recruited 28 patients with taste disorders (45–78 years) and 184 controls with no taste disorders (102 young [18–25 years] and 82 older [65–89 years] participants). Filter paper discs (5 mm dia.) were soaked in aqueous MSG solutions (1, 5, 10, 50, 100 and 200 mM), then placed on three oral sites innervated by different taste nerves. The lowest concentration participants correctly identified was defined as the recognition threshold (RT) for MSG. This test showed good reproducibility for inter- and intra-observer variability. We concluded that: (1) The RT of healthy controls differed at measurement sites innervated by different taste nerves; that is, the RT of the anterior tongue was higher than that of either the posterior tongue or the soft palate in both young and older individuals. (2) No significant difference in RT was found between young adults and older individuals at any measurement site. (3) The RT of patients with taste disorders was higher before treatment than that of the healthy controls at any measurement site. (4) The RT after treatment in these patients improved to the same level as that of the healthy controls. (5) The cutoff values of RT, showing the highest diagnostic accuracy (true positives + true negatives), were 200 mM MSG for AT and 50 mM MSG for PT and SP. The diagnostic accuracy at these cutoff values was 0.92, 0.87 and 0.86 for AT, PT and SP, respectively. Consequently, this umami taste sensitivity test is useful for discriminating between normal and abnormal umami taste sensations.

## Introduction

Umami taste has become known as the fifth basic taste but, for the following reasons, is different from the other four basic tastes (sweet, salty, sour, bitter) [1], [2]. In our taste clinic, some patients, especially older participants, have complained of a persistent subjective impairment of umami taste, although the other four basic taste sensations remained normal. This impairment sometimes remained even after clinical treatment had improved the other four basic taste sensations. Such patients often had complex problems including poor appetite and weight loss; observations consistent with previous findings indicating a close relationship between umami taste perception and physical condition in older people [3], [4].

To treat patients with taste disorders, their taste sensitivity must be reliably assessed, and indeed, some clinical taste tests using taste solutions are available and used routinely in the clinic for the four basic tastes [5]–[7]. However, a reliable umami taste sensitivity test that can precisely measure taste sensitivity at different sites in the oral cavity has yet to be established. Consequently, there is no detailed information regarding impaired umami taste sensitivity in patients with taste disorders.

The purpose of this study was therefore to develop and test a clinical umami taste sensitivity test that can discriminate between normal and abnormal umami taste sensation. To this end, we will investigate (1) whether umami taste sensitivity is different at different sites innervated by different taste nerves, (2) how umami taste sensitivity is distributed in healthy young and healthy older people, (3) if our method has sufficient intra- and inter-observer reproducibility, and (4) how umami taste sensitivity differs between healthy controls and patients with taste disorders.

## Materials and Methods

### Ethics statement

The present study was conducted according to the guidelines in the Declaration of Helsinki (http://www.wma.net) and was approved by a local ethics committee (the Ethics Committee of Tohoku University Graduate School of Dentistry, approved No. 2008/22-21). This study was carried out at Tohoku University Hospital between June 2009 and July 2012. Written informed consent was obtained from all participants.

### Inclusion and exclusion criteria

Healthy young and older individuals with no taste disorders were invited to participate in our project. The young healthy participants were students at our dental school and residents in our hospital. Older persons over 65 years old who lived in a home for senior citizens with daily meals arranged by a dietitian, as well as patients over 65 years old who visited our hospital with no complaint of a taste disorder, were included as healthy older participants. Individuals with hyposalivation or systemic diseases (e.g. endocrine, infectious or immunological diseases) were excluded as healthy controls. Salivation was assessed with the gum test (stimulated whole saliva collection method [8]–[9]), and a flow rate of <1.0 mL/min was considered as hyposalivation based on the proposal by the Research Committee on Sjögren's Syndrome of the Ministry of Health and Welfare in Japan [9]. Two hundred and sixty healthy controls (126 young and 134 older individuals) were initially asked to participate with 184 persons being suitable for our project (102 young [average age: 20.3±2.0 years, 18–25 years, 60 males and 42 females] and 82 older [average age: 76.9±5.3 years, 65–89 years, 20 males and 62 females] participants).

Thirty two patients, who visited our university hospital with a chief complaint of a taste disorder, also participated in this project. Those with a history of psychological problems were excluded after careful clinical interviews and psychological tests (Manifest anxiety scale [10] and Self-rating depression scale [11]) to avoid studying patients with psychogenic taste dysfunctions. Four patients were excluded as having a psychogenic taste dysfunction and thus 28 individuals (average age: 66.1±10.3 years, 45–78 years, 5 males and 23 females) were finally included as patients with taste disorders.

### Test solutions

Monosodium l-glutamate (MSG; monohydrate crystal; Ajinomoto Co. Inc., Kawasaki, Japan) was used as an umami taste substance. Six concentrations of MSG aqueous solution (1, 5, 10, 50, 100, and 200 mM) were prepared as umami test solutions using distilled water. The concentrations were selected according to previous findings [12]–[14] and our pilot study, which was conducted with our laboratory staff after training for chemosensory perception.

### Procedure for the umami taste sensitivity test

**Figure 1:**
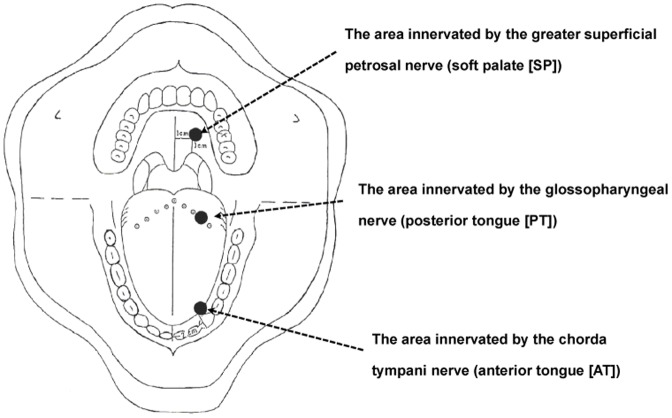
Measurement sites of the umami taste sensitivity test.

A filter paper disc (5 mm dia.) was soaked with an aqueous solution of MSG by pipetting a specific volume onto the disc and then placed with forceps on one of three specific sites that are innervated by different taste nerves: (i) the anterior tongue (AT), which is innervated by the chorda tympani nerve; (ii) the posterior tongue (PT), which is innervated by the glossopharyngeal nerve; and (iii) the soft palate (SP), which is innervated by the greater petrosal nerve (Fig. 1). This method is well established for measuring taste sensitivity for the four basic tastants (sweet, salty, sour and bitter) in Japan (Taste Discs™, Sanwa Chemical Laboratory Inc., Nagoya, Japan) [6]. One of the advantages of this method is using a wet paper disk which can be easily applied to patients with a dry mouth. All study participants were asked not to eat or drink anything except water, not to smoke, and not to brush their teeth for at least 1 hour before testing. Participants were familiarized in advance with the umami taste using a supra-threshold concentration of MSG before testing. To avoid any lingering taste, they carefully rinsed their mouths with distilled water until the taste sensation had disappeared.

The examiners, who administered the tests, were all dentists who had been trained in the taste sensitivity test with established guidelines and criteria. The participants were allowed to assess the taste for 3 seconds with their mouths open. The lowest concentration of MSG was always used first, and the concentration was increased sequentially until the umami taste was correctly recognized. The test solution with the lowest concentration that was correctly identified was defined as the recognition threshold (RT) for MSG. In accordance with the filter paper disc method established for the other four basic tastes, 1, 5, 10, 50, 100, and 200 mM MSG were defined as RT scores of 1, 2, 3, 4, 5, and 6, respectively. When the participant did not respond to the highest concentration of MSG (200 mM), RT was assigned a score of 7. Between each change in the MSG concentration or in the measurement site, participants were asked to rinse their mouths well with distilled water.

### Reproducibility of the test

For the assessment of intra-observer reproducibility, the umami taste tests were repeated by one well-trained examiner on a subgroup of 38 healthy participants (37.3±25.1 years, 18–81 years, 23 males and 15 females) at intervals from 1 to 8 days (mean interval, 5 days). To assess the inter-observer reproducibility, the umami taste tests were performed by five well-trained examiners on 31 healthy participants (22.1±2.0 years, 19–24 years, 21 males and 10 females).

The inter- and intra-observer variability of the RT value obtained from the umami taste tests were statistically analyzed to determine the reliability of this test.

### Treatment of patients with taste disorders

A treatment plan for each individual's taste disorder was devised based on clinical examinations including blood tests, salivary tests, an oral candida culture test and oral hygiene tests. In addition, patients were carefully interviewed regarding any systemic diseases and prescribed medications taking into account any side effects that could directly affect taste sensation.

### Statistical analysis

Because the distribution of the variables was non-normal, the Mann-Whitney U-test, based on ranking, was used to compare the continuous variables between two groups, i.e., the different measurement sites in the healthy young and older participants, and in the patient's variables before and after treatment. The criterion for significance was defined as p<0.05. SPSS 18.0 (SPSS Inc. Chicago, IL, USA) was used for the statistical analysis.

Intra- and inter-observer reliability for the umami taste test was assessed with Cohen's kappa statistic [15], [16] and with the Fleiss's kappa statistic [17], respectively, under weighted (quadratic) or unweighted conditions, using the Agreestat2011 software package (downloaded from http://www.agreestat.com/agreestat). The kappa coefficient adjusts for the proportion of agreement between or among observers by correcting for the proportion of agreement that could have occurred by chance. Perfect agreement results in a kappa value of 1.0: a kappa value of 0 indicates that the frequency of agreement was the same as that expected by chance alone. Interpretation of kappa coefficients was performed using Fleiss's benchmarking scale [18]. In short, a kappa value of 0.40 or less indicates a poor frequency of agreement between the observers, whereas values ranging from 0.4 to 0.75 and from 0.76 to 1.00 indicate intermediate-to-good and excellent levels of agreement, respectively.

To assess the diagnostic value of our umami taste sensitivity test, the receiver operating characteristic (ROC) analysis (patients before treatment vs. healthy older controls) was performed to find its sensitivity, specificity, positive predictive value (PPV), negative predictive value (NPV) and diagnostic accuracy (true positives + true negatives). The cutoff value that showed the highest diagnostic accuracy (the best cutoff value) to assess the subject as an umami taste disorder was also calculated from the ROC analysis for the umami taste sensitivity test. These diagnostic values comparing the AT, PT and SP sites were compared using Fisher's exact probability test. The ROC analysis was carried out using JMP 10 statistical software (SAS Institute Inc. Cary, NC, USA).

## Results

### Distribution of umami taste sensitivity at different measurement sites in healthy young and older participants

**Figure 2:**
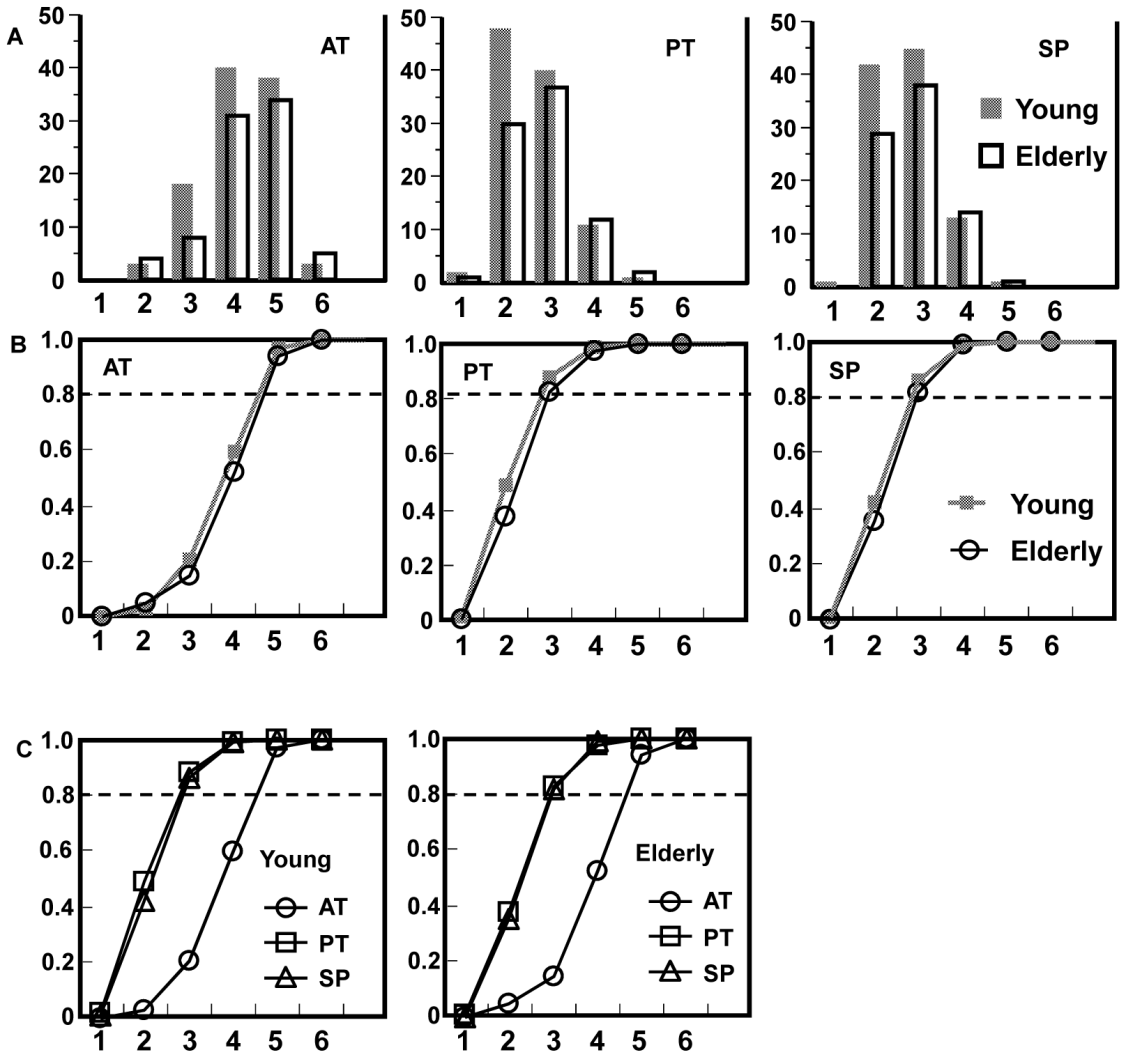
Distribution of umami taste sensitivity in healthy young and older persons at three different measurement sites. The abscissae shows the concentrations of the aqueous MSG solutions (1, 1 mM; 2, 5 mM; 3, 10 mM; 4, 50 mM; 5, 100 mM; 6, 200 mM). The ordinates shows the number of participants who correctly recognized MSG at each concentration of MSG (A), and the proportion of the participants who correctly recognized MSG the at each MSG concentration (B and C).

The distribution of umami taste sensitivity in healthy young and older controls for the three different measurement sites (Fig. 1), the anterior tongue (AT), the posterior tongue (PT) and the soft palate (SP), is shown in Figure 2A. The cumulative distribution for each measurement site (Fig. 2B) based on the data in Figure 2A indicates that ∼80% of the young and older controls showed an RT score of <3 (10 mM MSG) both for the PT (86.3% - young:, 81.7% - older) and the SP (85.3% - young:, 81.7% - older). However, the percentages of participants with an RT score of <3 for the AT were only 14.6% in older and 20.6% in young participants. The RT for the AT site was higher than for either the PT or SP sites in both young and older persons (Fig. 2C).

Table 1 shows the average score for RT in healthy young and older participants at the three different measurement sites. The RT score at the AT site was higher than those at the PT and SP sites, both in young and older participants (p<0.0001), whereas no significant difference was found between RT scores at the PT and SP sites for either young or older participants (p = 0.320 for young individuals and p = 0.722 for older individuals). In addition, there was no significant difference in the RTs between young and older persons at any of the three different areas innervated by taste nerves (AT, p = 0.220; PT, p = 0.104; SP, p = 0.264).

**Table 1:** Comparison of average scores of RT for MSG in healthy young adults and older participants at three different measurement sites, showing significance values.

Category	Site	RT Scores	Young-adult			Older-adult		
		Av±SD	AT	PT	SP	AT	PT	SP
Young-adult	AT	4.2±0.9						
	PT	2.6±0.7	<0.0001					
	SP	2.7±0.7	<0.0001	0.320				
Older-adult	AT	4.3±0.9	0.220					
	PT	2.8±0.8		0.104		<0.0001		
	SP	2.8±0.7			0.264	<0.0001	0.722	

Sites: AT: anterior tongue PT: posterior tongue SP: soft palate.

### Reproducibility of the test

Table 2 shows the intra-observer correlation based on Cohen's kappa value, the inter-observer correlation based on Fleiss's kappa statistic and the strength of agreement as defined by Fleiss [18]. The level of agreement for intra-observer reliability in each area ranged from “Intermediate to good” to “Excellent” under either unweighted or weighted conditions, indicating high reproducibility.

**Table 2:** Intra-observer reliability and inter-observer reliability.

	Intra-observer reliability (Cohen's Kappa)			Inter-observer reliability (Fleiss' Kappa)		
Site	unweighted	weighted	Strength of agreement	unweighted	weighted	Strength of agreement
anterior	0.77	0.86	Excellent	0.79	0.89	Excellent
posterior	0.73	0.75	Intermediate to good	0.87	0.90	Excellent
palate	0.75	0.81	Intermediate to good/Excellent	0.84	0.92	Excellent

Similarly, inter-observer correlations based on Fleiss's kappa statistic for the three different taste nerve–innervated areas were high. The level of inter-observer reliability in each area was “Excellent” under either unweighted or weighted conditions, showing high reliability.

### Differences in umami taste sensitivity between healthy controls and patients with taste disorders

Table 3 shows the changes in RT for MSG before and after treatment and the treatment target for each patient with a taste disorder. All 28 patients showed higher RT scores for MSG before treatment than after treatment. The mean RT scores before treatment were 6.3±0.7 for the AT, 5.5±1.2 for the PT and 5.4±1.2 for the SP. These values were higher than those for the healthy young and older controls (p<0.0001 for each measurement site). The RT scores after treatment were 4.1±0.6 for the AT, 2.5±0.6 for the PT, and 2.7±0.7 for the SP, significantly lower than those before treatment (p<0.0001 for each measurement site). In addition, the RT scores after treatment were not significantly different from those of healthy young persons (p = 0.455 for the AT, p = 0.450 for the PT and p = 0.788 for the SP) or older persons (p = 0.972 for the AT, p = 0.218 for the PT and p = 0.133 for the SP).

**Table 3:** Changes in the RT for MSG before and after treatment and the treatment target.

Patient	Age	Sex	Changes in recognition threshold (before →after)			Treatment target
	(years)		AT	PT	SP	
1	78	F	7 → 3	5 → 2	5 → 2	Anemia, Dry mouth, Oral candidosis
2	45	F	7 → 4	4 → 2	4 → 3	Anemia, Dry mouth, Oral candidosis, Oral mucositis
3	59	M	7 → 4	7 → 3	7 → 4	Cerebral infarct, Malnutrition
4	61	F	7 → 4	7 → 3	7 → 4	Common cold, Oral mucositis
5	70	M	7 → 5	7 → 3	7 → 3	Diabetes mellitus
6	69	F	7 → 5	7 → 4	7 → 4	Diabetes mellitus, Zinc deficiency
7	75	F	7 → 5	6 → 4	6 → 4	Diabetes mellitus, Zinc deficiency, Dry mouth
8	74	M	6 → 4	6 → 2	6 → 2	Diabetes, Dry mouth, Oral candidosis, Oral mucositis
9	54	F	5 → 4	4 → 2	5 → 2	Dry mouth, Oral mucositis
10	66	F	7 → 5	6 → 3	6 → 3	Gastritis, Oral candidosis
11	74	F	5 → 3	4 → 2	4 → 2	Gastritis, Oral mucositis
12	62	F	6 → 5	5 → 3	5 → 3	Gastritis, Oral mucositis
13	77	F	7 → 4	7 → 3	7 → 3	Gastritis, Zinc deficiency
14	75	F	6 → 4	4 → 2	5 → 2	Oral candidosis
15	75	F	6 → 4	5 → 2	5 → 2	Oral candidosis
16	77	F	6 → 4	5 → 3	5 → 3	Oral candidosis
17	60	F	6 → 4	6 → 3	4 → 3	Oral candidosis
18	67	M	7 → 4	7 → 2	7 → 3	Oral candidosis
19	50	F	5 → 4	4 → 2	4 → 2	Oral mucositis
20	56	F	6 → 4	5 → 3	5 → 3	Oral mucositis
21	68	F	6 → 5	5 → 2	5 → 2	Pernicious anemia, Humter's glossitis
22	66	F	5 → 3	4 → 2	4 → 2	Side effect of medication (anxiety), Dry mouth
23	45	F	6 → 3	6 → 2	5 → 2	Side effect of medication (pollen allergy), Zinc deficiency
24	65	F	7 → 4	4 → 2	5 → 2	Zinc deficiency, Dry mouth, Oral mucositis
25	49	M	7 → 4	7 → 2	7 → 3	Zinc deficiency, Oral candidosis
26	76	F	6 → 4	5 → 3	4 → 3	Zinc deficiency, Oral candidosis
27	76	F	7 → 4	7 → 2	7 → 2	Zinc deficiency, Oral candidosis, Oral mucositis
28	73	F	6 → 5	5 → 2	4 → 2	Zinc deficiency, Oral mucositis

RT: recognition threshold AT: anterior tongue PT: posterior tongue SP: soft palate.

### Diagnostic performance of the umami taste sensitivity test to assess umami taste disorder

The cutoff values of RT that showed the highest diagnostic accuracy (true positives + true negatives) were 6 for AT and 4 for both PT and SP. The numerical data are summarized in Table 4 which shows the area under the ROC curve (AUC), sensitivity, specificity, PPV, NPV and diagnostic accuracy. These diagnostic parameters showed no significant difference between the AT, PT and SP sites

**Table 4:** Diagnostic performance of the umami taste sensitivity test to assess the subject as an umami taste disorder.

	AT	PT	SP
AUC	0.95	0.97	0.97
Cut off value	6	4	4
Sensitivity	0.86	1.0	1.0
Specificity	0.94	0.83	0.82
PPV	0.83	0.67	0.65
NPV	0.95	1.0	1.0
Accuracy (TP + TN)	0.92	0.87	0.86

AT, anterior tongue; PT, posterior tongue; SP, soft palate;

AUC, area under the ROC curve; PPV, positive predictive value; NPV, negative predictive value; diagnostic accuracy  =  true positives (TP) + true negatives (TN).

## Discussion

### Advantage of this method for testing umami taste sensitivity

The main purpose of the present study was to develop an umami taste sensitivity test for clinical examination because detailed information for impaired umami taste sensitivity in patients with taste disorders has been unavailable. Various taste sensitivity tests have been developed, mainly for the four basic tastes (sweet, salty, sour and bitter) but not for umami. These methods include the whole-mouth method [12], [19], the taste strip method [20], the painting method [21] and the dripping method [5]. Of these methods, the whole-mouth method and the taste strip method have been used for assessing umami taste sensitivity. These methods are versatile and convenient to use but cannot assess the particular area that is impaired. The test solution used in the whole-mouth method spreads and almost immediately becomes diluted throughout the whole mouth. Consequently, the threshold obtained by this method might be because of the whole-mouth sensation. In contrast, umami taste sensitivity may differ in different areas of the mouth that are innervated by different taste nerves. The taste strip method, using dried filter paper strips that are soaked in different concentrations of the taste solution, has recently been used for the convenient measurement of taste sensitivity including umami taste [22]. With this method, participants lick the dried test paper with their tongues, and thus assessing the sensitivity at the PT area is difficult. In fact, the results of the strip method are not correlated with those obtained from an electrogustometry taste test that specifically examined the PT [23]. In addition, the dried taste strip may lead to an inaccurate assessment for patients with a dry mouth, because they may have difficulty dissolving the test substance in their saliva, which is required for stimulating taste receptors. In contrast to these methods, the filter paper disc method using a wet tastant can measure the threshold of a distinct area that is innervated by a specific taste nerve, such as the PT or the SP. In addition, the method is well established and widely used for assessing the four basic tastes. Therefore, we used the filter paper disc method for our umami taste sensitivity test.

### Umami taste solution and its concentration

We used MSG as an umami taste, as it is the most well-established substance that mediates the umami taste, being described a century ago by Ikeda [24]. The concentration of the umami tastant for the present study was based on information from a previous report which indicated that most healthy adults could recognize umami at a concentration of 80 mM MSG when placed on the PT area using the filter paper disc method [25]. We used quasi-half-log steps to provide a range of concentrations around the 80 mM MSG. The lowest concentration was set at 1 mM MSG based on the results [12] of the whole-mouth method which showed this value provided the lowest recognition threshold. Thus, the concentration of MSG was set at 1, 5, 10, 50, and 100 mM. In addition, going one–step higher, a concentration of 200 mM MSG was added for the patient with umami taste disorder. We estimated there would be a low risk for misidentifying the MSG umami taste as salty within our concentration range, because the highest MSG concentration (200 mM) in this study is still lower than the second lowest NaCl concentration (214 mM) used in the salt sensitivity filter paper disc test [6], a value below the threshold for the salty taste. In addition, our pilot study showed that an x% MSG solution (w/v) has the equivalent salty effect as an x/10 NaCl solution (unpublished data).

We first tried five MSG concentrations because the taste sensitivity tests for the four tastes use five concentrations each [6]. In our pilot study, we realized that the distribution of the RT for the AT shifted towards higher concentrations compared with those for the PT and SP. These different distributions for the different umami measurement sites differ from those for the other four basic tastes, which have similar thresholds and distribution curves regardless of their measurement sites [6], [25]. In our study, about 80% of the participants showed an RT in a range between 5 and 10 mM MSG for the PT and SP sites and in a range between 10 and 50 mM MSG for the AP.

When comparing the RT value for MSG with those from other studies, our concentration was a little higher than that from the whole-mouth method [19] and was considerably lower than from the taste strip method [22]. The RT from the whole-mouth method should be the total of the areas innervated by different taste nerves, whereas our filter paper disc method assessed only a small area. This may be the main reason why the RT from the whole-mouth method is lower than from our method. In contrast, the taste strip method uses dried strips with the tastant, and thus a higher concentration of MSG may be needed to diffuse in the saliva and reach the taste buds. Indeed, the MSG concentrations for the taste strip method as used by Mueller et al. [22] were ∼6 times higher than concentrations in the present study. This higher RT from the taste strip method compared with the filter paper disc method is also seen with the sweet, salty, sour and bitter tastes [22].

### Regional difference in umami sensitivity

The RT for the PT and SP sites was lower than that for the AT in healthy young and older individuals (Table 1). This agrees with previous findings by Yamaguchi [25], who measured a lower sensitivity to MSG for the AT. Halpern [26] also reported that stimulating the anterior part of the human tongue with MSG did not induce umami taste perception but rather resulted in the perception of combinations of the four basic tastes, whereas the posterior part of the tongue elicited the umami taste. These regional differences within the oral cavity are supported by previous electrophysiological findings in several species of mammals showing that the glossopharyngeal nerve, which innervates the foliate and vallate papillae of the PT, contains fibers that are highly sensitive and selective for umami taste substances [27]. Such umami taste–sensitive fibers are not observed in the chorda tympani nerve, which innervates the fungiform papillae of the AT. Thus, we have demonstrated that the rear of the oral cavity is more sensitive to umami taste than the tip of the tongue.

Recent progress in molecular biology has identified several umami taste receptor candidates, such as the heterodimer taste receptor type 1, members 1 and 3 (T1R1/T1R3) [2], [28] and brain-expressed and taste-expressed type 1 and 4 metabotropic glutamate receptors (brain and taste mGluR1 and mGluR4) [1], [29]–[31]. The accumulating evidence from mice indicates that the potential role of the signal mediated by the transduction pathway involving T1R1/T1R3 may be different from that mediated by the pathway involving the mGluRs. The former signal occurs mainly in the AT and plays a major role in preference behavior, whereas the latter occurs mainly in the PT and is active in mice lacking T1R3, contributing to behavioral discrimination between umami taste and other taste compounds [32]. In humans, unlike in mice, T1R1/T1R3 acts as an umami taste–specific receptor that can discriminate between umami taste and other tastes and thus accounts for umami-linked preferences and discrimination [32]. Further studies are essential for determining the specific roles of T1R1/T1R3 in high sensitivity to umami taste in the human PT as observed in the present study.

### Difference in umami taste sensitivity between the young and older people

We observed no significant difference in the RTs between young and older participants at any of the three different measurement sites (Table 1). This may indicate that our umami taste sensitivity test can be used for both young and older individuals at the same cut-off point to discriminate between normal and impaired taste sensitivity. In contrast, Schiffman et al. [12] reported that the RT for an umami tastant (glutamate salts) is higher in older people than in the young when measured with the whole-mouth method. They suggested that a decrease in the number of taste buds may be related to taste disturbances in older people. However, the few studies on taste buds in aged mammals have been contradictory: two studies reported taste bud loss in circumvallate papillae of aged human and murine tongues [33], [34]: another study reported no change in taste bud numbers in human fungiform papillae throughout life, from birth to old age [35]. Mistretta and Baum [36] reported no difference in the number or size of taste buds in the papillae in the AT and PT between young and old rats. This study also reported that both anatomical investigations and human taste threshold studies indicated that age-related differences in the gustatory system are not as substantial as investigators have suggested in the past [36]. This idea complements our results in demonstrating no difference in umami taste sensitivity between young and older participants. In the present study, we carefully excluded individuals with hyposalivation or systemic diseases (e.g., endocrine, infectious and immunological diseases), although most previous reports examining human taste thresholds did not consider the general condition of the participants. We paid particular attention to individuals with a dry mouth because this condition is common in the older and is strongly related to taste disorders [37]. Following measurements of the salivary secretion rate, we excluded older persons with a dry mouth from this study, even though some of these individuals have no subjective feeling of oral dryness. Consequently, we were able to obtain a similar level of umami taste sensitivity in healthy young and older persons, indicating that our sensitivity test can be clinically applied using common criteria for both populations.

### Diagnostic performance of our test for discriminating between normal and abnormal umami taste sensation

To apply this umami taste sensitivity test for clinical use, the reproducibility of the test is essential. In this respect, our test showed high reliability for intra- and inter-observer variability as determined by the kappa statistic (Table 2).

Based on our basic data concerning umami taste sensitivity in healthy individuals, we measured umami taste sensitivity in patients with a taste disorder. To select and identify patients with a taste disorder, we excluded those with a history of psychological problems, because such patients may show difficulty in responding to the taste sensitivity test, based on a report indicating that gustatory hallucinations can occasionally be observed in various psychotic disorders [38]. Our results have shown that the mean RT scores for MSG in patients with a taste disorder before treatment were higher than those in healthy individuals at every measurement site (AT, PT and SP). After treatment, the MSG RT scores for the patients reached the same levels as those from the healthy controls at each measurement site, indicating that a loss of umami taste sensitivity can be improved with appropriate treatment. Regarding the treatment target, we paid attention to dry mouth, oral candidosis, oral mucositis and systemic diseases as causal factors in taste disorders (Table 3). These clinical results indicated that the RT before, during and after treatment can be assessed with an umami taste sensitivity test using MSG. Finally, we compared the diagnostic performance of our test to decide whether the subject had an umami taste disorder by analyzing sensitivity, specificity, PPV, NPV and diagnostic accuracy (true positives + true negatives). The cutoff value of RT that showed the highest diagnostic accuracy (true positives + true negatives) was calculated from the ROC analysis, which resulted in 6 for AT and 4 for both PT and SP. As shown in Table 4, the diagnostic performance of our umami taste sensitivity test was high, indicating that the test is useful for discriminating between normal taste and an umami taste disorder using these cutoff values. In addition, Table 4 shows that these diagnostic parameters did not differ significantly at each measurement site.

Until recently, umami taste dysfunction had not been fully recognized and detailed information about impaired umami taste sensitivity in patients with a taste disorder had not been described: this is the first report showing the existence of patients with an umami taste disorder. It is of particular importance that all patients who complained of a taste disorder showed higher RT scores for MSG before treatment. We did not examine the RT for the other four basic tastes in the present study, but we could assume that umami taste sensitivity is closely related to the subjective feeling of taste loss. Decreased taste sensitivity may induce appetite loss and health problems, resulting in a lower quality of life, particularly in the older. Thus, this umami taste sensitivity test may be essential for treating patients with a taste disorder.

## Supporting Information

Figure S1 Histograms (upper part) showing distribution of the umami sensitivity for the patients before treatment (red) and the controls (blue). Abscissae: concentrations of the aqueous MSG solutions (1, 1 mM; 2, 5 mM; 3, 10 mM; 4, 50 mM; 5, 100 mM; 6, 200 mM; 7, >200 mM). Ordinates: number of the participants who correctly recognized MSG at each concentration of MSG, and the ROC curves (lower part) to assess the diagnostic value of the umami taste sensitivity test (See Table 4). AT, anterior tongue; PT, posterior tongue; SP, soft palate.
(PDF)
