# Supplementary figures and images for: Development of an Umami Taste Sensitivity Test and Its Clinical Use
Source: PLoS One. 2014 Apr 18;9(4):e95177. doi: 10.1371/journal.pone.0095177 (PMC3991614; doi:10.1371/journal.pone.0095177)

Number of  
subjects

**AT**

Number of  
subjects

**PT**

Number of  
subjects

**SP**

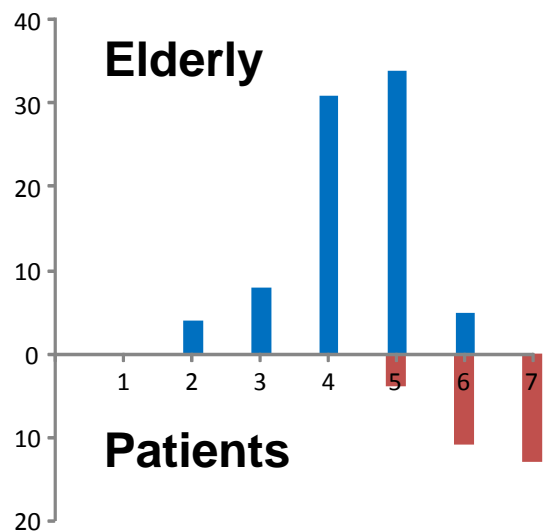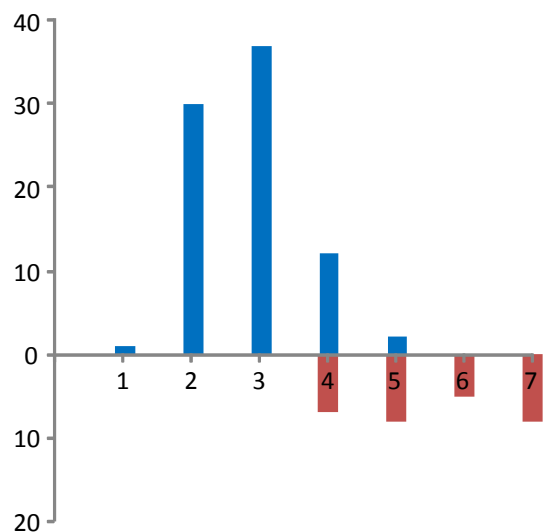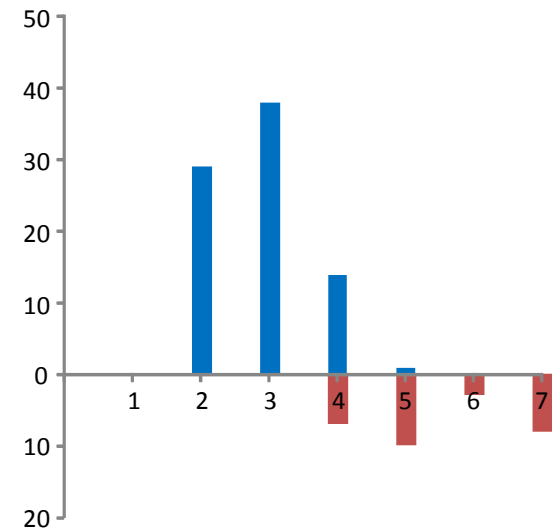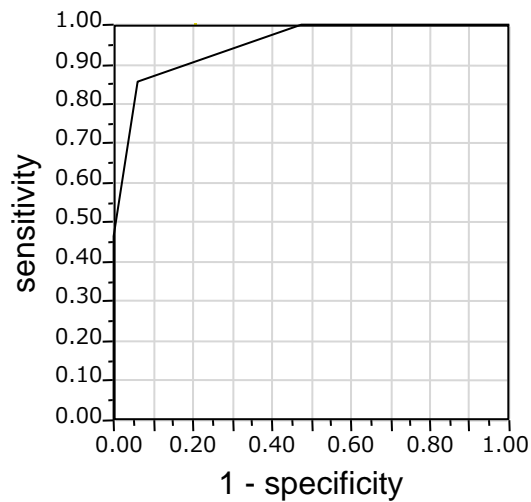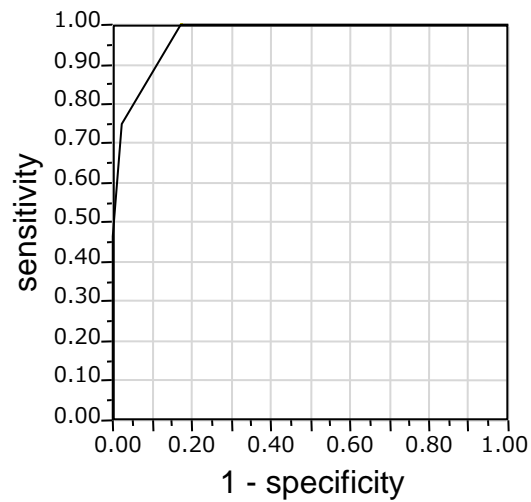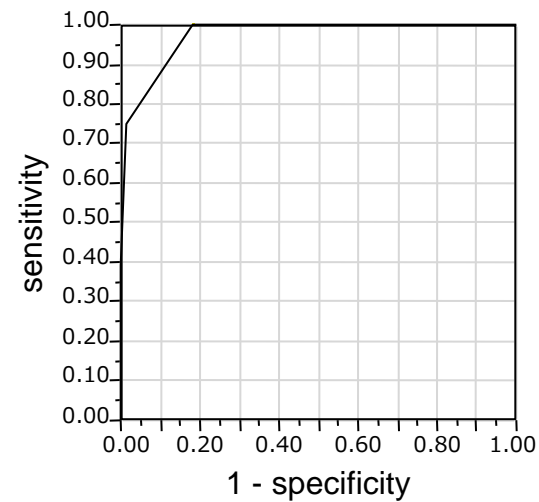

Supplement: Figure S1 — Histograms (upper part) showing distribution of the umami sensitivity for the patients before treatment (red) and the controls (blue). Abscissae: concentrations of the aqueous MSG solutions (1, 1 mM; 2, 5 mM; 3, 10 mM; 4, 50 mM; 5, 100 mM; 6, 200 mM; 7, >200 mM). Ordinates: number of the participants who correctly recognized MSG at each concentration of MSG, and the ROC curves (lower part) to assess the diagnostic value of the umami taste sensitivity test (See Table 4). AT, anterior tongue; PT, posterior tongue; SP, soft palate. (PDF) [file pone.0095177.s001.pdf]
